# Primary Umbilical Endometriosis: Unusual and Rare Clinical Presentation

**DOI:** 10.1155/2016/9302376

**Published:** 2016-05-08

**Authors:** Fuminori Taniguchi, Eriko Hirakawa, Yukihiro Azuma, Chihiro Uejima, Keigo Ashida, Tasuku Harada

**Affiliations:** ^1^Department of Obstetrics and Gynecology, Tottori University Faculty of Medicine, Yonago 683-8504, Japan; ^2^Department of Surgery, Division of Surgical Oncology, Tottori University Faculty of Medicine, Yonago 683-8504, Japan

## Abstract

Primary umbilical endometriosis is a rare disorder and is defined as the presence of ectopic endometrial tissue within the umbilicus. A patient with painful mass in the umbilicus during menstrual period is studied in this paper. The possibility of subcutaneous endometriosis should be considered when an umbilical mass is detected despite the absence of previous surgery. In this case, urachal cancer, urachal remnant, umbilical endometriosis, and its malignant transformation were among the diseases considered in the differential diagnosis. Complete excision and histology are necessary to obtain a definitive diagnosis and optimal treatment for umbilical subcutaneous endometriosis.

## 1. Introduction

Endometriosis is defined by the presence of endometrial tissue outside the uterus. The precise rate of prevalence of umbilical endometriosis is not known, but primary umbilical endometriosis is a rare disorder. The incidence of this disease is estimated to be about 0.5% to 1% of all cases of extragenital endometriosis [1]. Although the pathogenesis of endometriosis is still obscure, several theories including retrograde menstruation, direct spread, embryonal rest, coelomic metaplasia, and lymphatic or hematogenous spreading are thought to be possible causes of the development of endometriosis.

Cyclical pains are coincidental with a tumor, and palpable masses are the most common symptoms of primary umbilical endometriosis. Although the pathogenesis of this disease is not fully understood, possibilities include the migration of endometrial cells to the umbilicus through the abdominal cavity or the lymphatic system or embryonic remnants in the umbilical fold such as the urachus and the umbilical vessels [1, 2]. In contrast, secondary umbilical endometriosis is caused by iatrogenic dissemination of the eutopic endometrial cells after surgery [2, 3]. The iatrogenic implantation occurs in the surgical scars including cesarean section, laparoscopic surgery, and episiotomy. The cause of primary umbilical endometriosis cannot be explained by this theory.

The patient considered here has a rare primary umbilical endometriosis and had been diagnosed with urachal cancer. In this case, complete surgery and histopathological examination were essential as the appropriate treatment.

## 2. Case Report

Our subject was provided with written informed consent with guarantees of confidentiality. She was a 45-year-old multiparous patient with a painful umbilical mass concomitant with menstruation. She had no past history of any surgery and her medical histories were unremarkable. She has been complaining of progressive dysmenorrhea 3 years ago, and it was diagnosed as endometriosis based on her clinical symptoms by a gynecologist in another hospital. At that time, the patient did not show a pigmented area in the umbilical nodule. Initially, she took Dienogest (DNG), a progestin, for 14 months. Subsequently, while on treatment with oral contraceptives (OC) for 18 months, she felt a gradual increase in size of the subcutaneous induration and more tenderness around her umbilicus.

**Figure 1:**
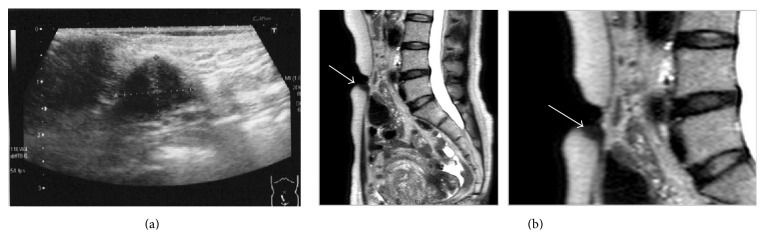
(a) Transcutaneous USG and (b) abdominal MRI (T2-weighted sequence). USG showed 1.0 × 1.5 cm umbilical nodule. MRI also exhibited the hypodense umbilical nodule (arrow) and myoma (asterisk).

**Figure 2:**
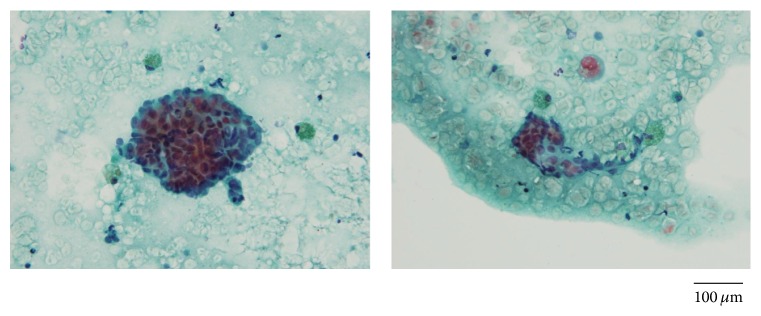
The specimens of fine needle aspiration biopsy. Bar: 100 *μ*m.

A physician in another clinic performed ultrasonography (USG) and fine needle aspiration biopsy (FNB). At that time, USG and MRI (magnetic resonance imaging) revealed about 1.0 × 1.5 cm solitary nodule in her caudal umbilicus (Figure 1), and adenocarcinoma was suspected by cytodiagnosis. The specimens from the FNB showed two lesions aggregated with adenomatous cells having a high nucleus/cytoplasm ratio and atypical cells, and macrophages laden with hemosiderin were found (Figure 2). A PET-CT (positron emission tomography-computed tomography) showed a slight accumulation of FDG (fluorodeoxyglucose). But the pathologist could not diagnose definitively whether or not this was an abnormal finding and ruled out the possibility of metastatic carcinoma.

The patient was subsequently introduced to the department of surgery in our hospital and was diagnosed with suspicious urachal cancer. She then visited our department as an outpatient. This nodule was a nonbleeding rigid mass and discolored, which could not be reduced by digital pressure. Using transvaginal USG, a myoma (4 cm in diameter) at the fundus of the uterus was found. At that time, abdominal MRI did not reveal a lesion of pelvic endometriosis. Serum CA125 and SCC values were within normal ranges. There was no sign of infection and bleeding in the umbilical lesion.

**Figure 3:**
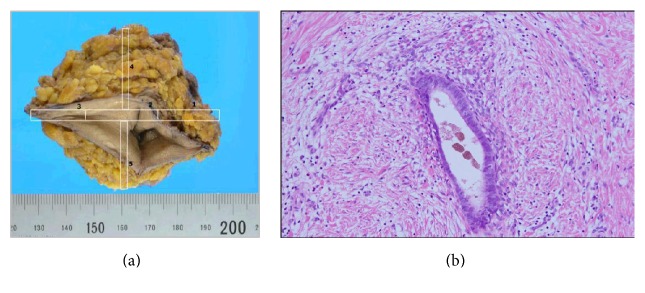
(a) Excised specimen of the umbilicus with surrounded tissue. (b) Histological microscopic view of this lesion demonstrated endometrial gland and stroma (H&E staining: ×10).

Based on the diagnosis of urachal cancer, urachal remnant, umbilical endometriosis, or its malignant transformation, we excised surgically the rigid lesion together with umbilicus and its surrounding tissues consisting of skin, fat, and fascia under general anesthesia (Figure 3). Histopathological examination of the specimens revealed it as an umbilical endometriosis consisting of endometrial glands. No malignancy was identified. She has used OC cyclically after the surgery. After 1 year of follow-up, she has not had any complaints, with no sign of recurrence.

## 3. Discussion

Among all diagnosed endometrioses, 1% to 12% of patients have them at extragenital sites, such as the lungs, diaphragm, or umbilicus [4, 5]. Primary umbilical endometriosis is a rare entity but should be taken into account in differential diagnosis of umbilical disorders even in women with no typical symptoms of pelvic endometriosis. The umbilicus is a preferred spot for cutaneous endometriosis; however, only 3 cases of primary umbilical endometriosis surgically confirmed not to have endometriotic lesions in the abdominal cavity have been reported in previous literature [6–8]. The theory of lymphatic or hematogenous spreading is favored with primary umbilical endometriosis, although the etiology of umbilical endometriosis is not completely understood.

Primary umbilical endometriosis is typically manifested by a firm, pigmented, or bluish nodule with pain and tenderness associated with cyclic bleeding or discharge during menstruation. In this subcutaneous nodule, the patient did not show any skin symptoms, pigmentation, or bleeding in the umbilicus. It was impossible to establish a definitive diagnosis of umbilical endometriosis by only the USG or MRI findings. Zhai also stated that the FNB is a useful additional tool for diagnosing cutaneous endometriosis [9]. Although there are no reports on the diagnostic accuracy of FNB in umbilical endometriosis, Zhao et al. stated that undiagnostic aspiration rate of FNB is as high as 75% for the abdominal endometriosis including the umbilicus endometriosis [10]. In this case, the diagnosis was difficult in spite of using USG, MRI, and FNB.

Hormonal therapy, such as DNG or OC, was effective preoperatively in ameliorating the symptoms of this disease but could not completely control the pain of the umbilicus lesion. Surgical excision is most frequently used as a safe and definitive treatment of umbilical endometriosis [1, 11]. When a malignancy such as urachal cancer or its malignant transformation of umbilical endometriosis could not be denied, complete excision should be carried out.

The risk of malignant transformation from umbilical endometriosis is very low. Only two cases of umbilical endometrioma with malignant transformation have been reported thus far. Lauslahti first reported a case of adenocarcinoma of umbilical endometriosis in 1972 [11]. Obata et al. also described a patient with endometriosis adjacent to a clear cell carcinoma that transformed into a carcinoma from endometriosis at the umbilical lesion [12].

The urachus is the main excretory organ of the fetus located in a region from umbilicus to upper bladder and is present in all children at birth and then gradually degenerates into a single fibrous band connecting the umbilicus to the dome of bladder after birth. Although we considered urachal cancer as a different diagnosis, it is a rare form of cancer that can sometimes involve the bladder. Urachal cancer stems from malignant transformation of the remaining enteric epithelium in the urachus. Currently, there is no consensus on the diagnostic criteria for urachal cancer, and it is considered to have no specific symptoms. Urachal cancer is generally aggressive, appears at an advanced stage, and has poor prognosis. Surgery, including partial cystectomy and radical resection [13], is the treatment of choice for this disease.

In summary, primary umbilical endometriosis is a rare and underrecognized phenomenon. Nevertheless, this disease must be considered in the differential diagnosis upon examining any umbilical lesions. Complete excision with successive histology is recommended for obtaining a definitive diagnosis and optimal treatment.
